# The first structure in a family of peptidase inhibitors reveals an unusual Ig-like fold

**DOI:** 10.12688/f1000research.2-154.v2

**Published:** 2013-08-23

**Authors:** Daniel J Rigden, Qingping Xu, Yuanyuan Chang, Ruth Y Eberhardt, Robert D Finn, Neil D Rawlings

**Affiliations:** 1 Institute of Integrative Biology, University of Liverpool, Liverpool, L69 7ZB, UK; 2 Joint Center for Structural Genomics, La Jolla CA, 92037, USA; 3 Stanford Synchrotron Radiation Lightsource, SLAC National Accelerator Laboratory, Menlo Park CA, 94025, USA; 4 Sandford-Burnham Institute, La Jolla CA, 92037, USA; 5 Wellcome Trust Sanger Institute, Wellcome Trust Genome Campus, Hinxton, CB10 1SA, UK; 6 European Molecular Biology Laboratory, European Bioinformatics Institute,Wellcome Trust Genome Campus, Hinxton, CB10 1SD, UK; 7 Howard Hughes Medical Institute, Janelia Farm Research Campus, Ashburn VA, 20147, USA

**Keywords:** X-ray crystallography, protein structure, PDB, peptidase, inhibitor, Bacillus subtilis, Ig fold

## Abstract

We report the crystal structure solution of the Intracellular Protease Inhibitor (IPI) protein from *Bacillus subtilis*, which has been reported to be an inhibitor of the intracellular subtilisin Isp1 from the same organism. The structure of IPI is a variant of the all-beta, immunoglobulin (Ig) fold. It is possible that IPI is important for protein-protein interactions, of which inhibition of Isp1 is one. The intracellular nature of ISP is questioned, because an alternative ATG codon in the *ipi* gene would produce a protein with an N-terminal extension containing a signal peptide. It is possible that alternative initiation exists, producing either an intracellular inhibitor or a secreted form that may be associated with the cell surface.  Homologues of the IPI protein from other species are multi-domain proteins, containing signal peptides and domains also associated with the bacterial cell-surface. The cysteine peptidase inhibitors chagasin and amoebiasin also have Ig-like folds, but their topology differs significantly from that of IPI, and they share no recent common ancestor. A model of IPI docked to Isp1 shows similarities to other subtilisin:inhibitor complexes, particularly where the inhibitor interacts with the peptidase active site.

## Introduction

The Isp1 peptidase (also known as IspA) is the major intracellular peptidase in *Bacillus subtilis*, accounting for more than 80% of the intracellular azocasein- or azocollagen-degrading activity ^1^. It is a subtilisin homologue and a member of peptidase family S8. It is unclear what the physiological role of Isp1 is; it has been shown not to be important during sporulation where the majority of intracellular proteolysis occurs, even though intracellular proteolysis was correspondingly low when a defective mutant of Isp1 was created ^2^. Potential physiological substrates that have been identified include elongation factor Tu (cleavage within the GTP binding domain, PF00009) and ClpC, a negative regulator of genetic competence (with one cleavage occurring in a disordered region and another in a coiled-coil region) ^3^. The peptidase is synthesized as a precursor with an N-terminal, 18-residue propeptide that blocks the active site. Release of the propeptide is probably by Isp1 itself, inter-molecularly ^4^. The structures of both the precursor and the mature peptidase have been solved and show that a proline residue (Pro8) introduces a kink in the backbone that shifts the scissile bond in the propeptide away from the catalytic serine. Once the propeptide is released, the peptidase active site forms correctly ^4^.

The activity of a powerful endopeptidase within the cell must be controlled in order to prevent unwanted proteolysis of cellular proteins, and in 1986 Nishino *et al.* ^5^ described an inhibitor of Isp1 from *Bacillus subtilis* known as Intracellular Protease Inhibitor (IPI). This inhibitor was effective against a number of subtilisin homologues, using casein as a substrate following pre-incubation of peptidase and inhibitor, but was ineffective against cysteine, aspartic and metallopeptidases. A *K*_i_ for inhibition of the Isp1 peptidase was estimated to be in the order of 10 ^-9^ M ^6^. The gene for the peptidase has been cloned ^7^, and the deduced protein sequence showed, at the time, no similarity to any other protein. There are now 149 homologues in the Pfam family PF12690 (BsuPI). The family was included in the MEROPS database (as I22 ^8^), but subsequently removed, because it was not clear whether the proteins in the family were inhibitors or competing substrates. In particular, the inhibition of intracellular peptidases Isp2 and Isp3 was “repressed” in the presence of the substrate casein ^6^, which suggests the activity of competing substrates.

Structural similarity to other known peptidase inhibitors might argue for reinstatement of the family in the MEROPS database. However, most peptidase inhibitors are secreted and intracellular peptidase inhibitors are rare. The only examples are cystatins A and B from family I25, which inhibit cysteine peptidases ^9^; calpastatin, which inhibits the intracellular peptidase calpain ^10^; chagasin from the zooflagellate *Leishmania* (family I42), which also inhibits cysteine peptidases ^11^; three intracellular coagulation inhibitors from the horseshoe crab *Tachypleus* that are serpins from family I4 ^12^; and pinA from family I24, which is an inhibitor of the ATP-dependent serine endopeptidase Lon, but is of unknown structure ^13^. Despite the inhibitors being intracellular, the known target peptidases are all extracellular, with the exceptions of calpain and endopeptidase Lon. Given the paucity of known intracellular peptidase inhibitors, it would not be a surprise if the fold of IPI were different from any known inhibitor structures, especially the secreted inhibitors which are stabilized by disulfide bridges, because intracellular proteins lack disulfides.

Most serine peptidase inhibitors act as if they were super-substrates, binding so tightly to the active site that they are either not cleaved, or if cleavage occurs then the fragments are not released from the peptidase. There is a bond, known as the reactive bond, which occupies the peptidase active site with residues either side occupying the S1 and S1′ binding pockets (in the nomenclature of Schechter & Berger, 1968 ^14^). This inhibitory mechanism is known as the standard or the Laskowski mechanism ^15^. The residues that form the reactive bond will vary from inhibitor to inhibitor, according to the specificity of the peptidase that is inhibited. The chymotrypsin-like specificity of the Isp1 peptidase implies that the P1 residue in the reactive bond of the intracellular inhibitor should be a hydrophobic residue.

A preliminary NMR study, assigning chemical shifts to the *B. subtilis* intracellular peptidase inhibitor has been published ^16^, which identified beta strands. We report the complete tertiary structure of the intracellular peptidase inhibitor from *Bacillus subtilis*.

## Methods

### Structural determination

The American Type Culture Collection (ATCC) provided the genomic DNA used to clone *ipi* (ATCC Number: 23857D-5). Protein production and crystallization of IPI was carried out by standard JCSG protocols ^17^. The crystal was obtained using the vapor diffusion method in a sitting drop format where sitting drops composed of 100 nl protein solution mixed with 100 nl crystallization solution were equilibrated against a 50 μl reservoir at 293 K. The crystallization reagent consisted of 48.5% polyethylene glycol 600, 0.1M CHES pH 9.7. Data were collected at wavelengths corresponding to the inflection and high energy remote of a selenium MAD (multi-wavelength anomalous dispersion) experiment at 100 K using a MARCCD 325 detector (Rayonix) at Stanford Synchrotron Radiation Lightsource (SSRL) beamline 9_2. Data processing were carried out using XDS ^18^ and the statistics are presented in Supplementary table 1. The structure was determined by the MAD method using programs SHELX ^19^ and autoSHARP ^20^, and refinement was carried out using REFMAC5 ^21^. The structure was validated using the JCSG Quality Control server ( http://smb.slac.stanford.edu/jcsg/QC). Atomic coordinates and experimental structure factors to 2.6 Å resolution (PDB code: 3ISY) have been deposited in the Protein Data Bank ( www.pdb.org ^22^).

### Bioinformatics

Sequence conservation among homologues of IPI was mapped onto the crystal structure using ConSurf ( http://consurf.tau.ac.il/; ^23^). The results were visualised with PyMOL ( http://www.pymol.org/), which was also used for structural figures.

A homology model of *B. subtilis* intracellular proteinase, IspA, was created at the Swiss-model server ^24^ using the structure of processed, active intracellular protease from *Bacillus clausii* (PDB code 2XRM), around 50% identical to IspA, as the sole template. The high degree of sequence identity with the target and the small number of insertions and deletions between target and template (one six residue deletion and a single insertion of one residue, both readily accommodated) assured a high quality model. Additionally, the insertions and deletions lie distant from the catalytic site which was the main region of interest.

The new structure of IPI was docked to the model of IspA using three different webservers, GRAMM-X ( http://vakser.bioinformatics.ku.edu/resources/gramm/grammx/; ^25^), ZDOCK ( http://zdock.umassmed.edu/; ^26^) and ClusPro 2.0 ( http://cluspro.bu.edu/; ^27^).

## Results and discussion

### Structure description

The crystal structure of an intracellular proteinase inhibitor (IPI, gene locus BSU11130) from *Bacillus subtilis* was determined to 2.6 Å resolution by the MAD method. Data collection, model and refinement statistics are summarized in Supplementary table 1. The final model includes one molecule (residues 3–119), one tetraethylene glycol and 21 water molecules in the asymmetric unit. The structure is mainly composed of nine beta strands. Gly0 (which remained at the N-terminus after cleavage of the expression/purification tag), Met1 and Glu2 were disordered and not modeled. All the side chains except for Glu17, Lys60 and Lys97 were fully modeled. The Matthews coefficient ( *V _M_* ^28^) is 3.2 Å ^3^ Da ^-1^ and the estimated solvent content is 60.9%. The Ramachandran plot produced by *MolProbity* ^29^ shows that 98.0% of the residues are in favoured regions, with no outliers. Two residues, Lys 86 and Glu 87, are flagged as having unusual Ramachandran plot properties by the PDB validation report although not with Molprobity. Electron density for them strongly supports their modelled conformations.

Validation report for PDB: 3ISY This is the first structure in a family of peptidase inhibitors derived from Bacillus subtilis. The structure has an unusual Ig-like fold.

**Figure 1.**
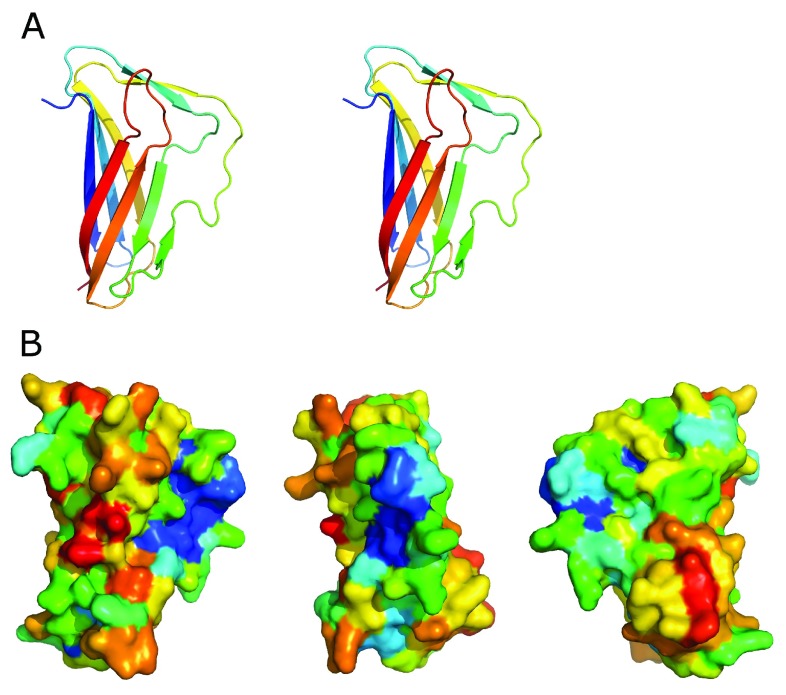
Overall structure of Intracellular Protease Inhibitor (IPI) reveal a wedge shape with a conserved surface. **A**) Stereo cartoon representation of IPI coloured from blue to red, N- to C-terminus. Protruding loops on the right hand side form a wedge shape. **B**) Sequence conservation amongst known homologues mapped to the protein surface with red indicating high conservation and blue low. The left panel shows IPI in the same orientation as in **A**), the other two show the results of successive 90 degree rotations about a vertical axis so that the wedge is seen end-on in the centre panel and the right panel shows the opposite face to the left panel.

The structure of the IPI protein is similar to the immunoglobulin (Ig) fold. This common fold consists of a beta-sandwich formed of seven strands in two sheets with a Greek-key topology and proteins with this fold are involved in a variety of functions, including cell-cell recognition, cell-surface receptors, muscle structure and the immune system. The limits of the B-strands seen in the structure coincide well with those determined by NMR ^16^ ( Supplementary table 2). At the strand level there is only a single disagreement: residues 62–65 were assigned as B-6 by NMR but in the crystal structure form an extended region that lacks the backbone hydrogen bonding characteristics to be defined by us as a strand. The structure of IPI shows an unusually extended loop on one edge of the Ig fold, seen on the right hand side in Figure 1A, resulting in a wedge-like or prismatic overall shape. As Figure 1B shows, this unusual feature is conserved at the sequence level among members of the I22 family, suggesting that it may harbour a functional site.

From a comparison using the Dali website ( http://ekhidna.biocenter.helsinki.fi/dali_server/ ^30^) the protein with the closest structural similarity is the RbmA protein from *Vibrio cholerae* (Z-score 10.0, Root-Mean-Square Deviation (RMSD) 2.9 Å, residues 38–152) *,* which is one of the three major protein components of the biofilm matrix important for cell-to-cell contacts ^31^. The A chain from coagulation factor XIII ^32^ is also structurally similar, with z-scores in the range of 9.4–9.9 depending on the source species. The structural similarity with IPI is over residues 518–629 for human factor XIII, which is a domain from the transglutaminase, C-terminal Ig-like domain family ( PF00927). Factor XIII is a transglutaminase important for stabilizing fibrin clots by cross-linking chains with isopeptide bonds ^32^. These similarities probably reflect only the all-beta nature of the structures rather than any common physiological functions.

**Figure 2.**
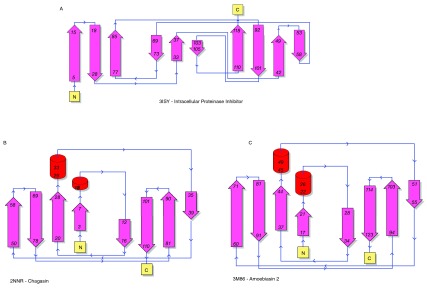
Secondary structure topologies of Intracellular Protease Inhibitor (IPI) and other peptidase inhibitors with an Ig-like fold. Topology diagrams are from PDBSum ^43^. The labels indicate the PDB accession number and the protein name.

The folds of chagasin and amoebiasin (both members of inhibitor family I42) have also been described as Ig-like ^33, 34^. From a Dali pairwise comparison, the structures of IPI and chagasin (PDB code 2NNR) are similar but distant (Z-score 2.2, RMSD 4.2 Å; structural alignment over 110 residues). The structures of IPI and amoebasin (PDB: 3M86) are also similar (Z-score 3.0, RMSD 4.5 Å, over 111 residues). Figure 2 shows a secondary structure comparison of IPI, chagasin and amoebiasin. Chagasin and amoebiasin have similar topologies, but the order of the beta strands differs significantly between these two I42 family members and IPI. This implies that IPI is not closely related to the I42 family, and that the similar folds may have been acquired independently by convergent evolution.

### Docking

In order to explore the hypothesis that IPI is an inhibitor of the intracellular protease IspA, structure docking was carried out. As an experimental structure for *B. subtilis* was not available, a homology model was created. The ~50% sequence identity shared with available templates assures a high quality model: at this level of similarity an RMSD error of around 1 Å is expected ^35^. Since it is often difficult to pick out the true solution from the multitude of poses typically resulting from a docking experiment, we applied three cross-checks to improve confidence in our results. First, we required that the interface of the inhibitor with protease involve the conspicuously conserved wedge structure ( Figure 1B), which is indicated as the functional site of the inhibitor structure. We chose not to incorporate this information in the docking runs, although several servers allow this, in order to reserve it as an independent check on the results. Secondly, we required that a solution sterically block the catalytic site cleft since this, rather than allosteric mechanisms, is the predominant mode of protease inhibition. Thirdly, we sought docking poses that were independently proposed by different algorithms; although simple structural compatibility is a principal criterion for all docking servers, they differ in their scoring functions so that poses jointly flagged by different methods may be considered more reliable.

**Supplementary figure 1.**
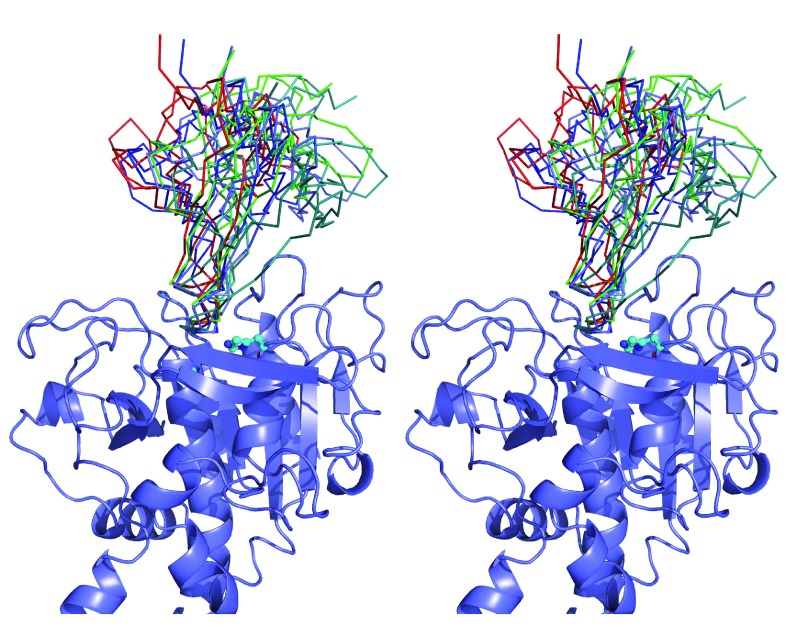
Stereo view of the superposition of docking poses from ZDOCK (numbers 1, 4 and 5, coloured shades of blue), GRAMM-X (number 2, coloured red) and ClusPro (number 3, coloured green) showing broadly similar modes of inhibitor interaction with the enzyme, shown as cartoon below with catalytic triad drawn in ball-and-stick.

Comparison of the top 10 results from each of the docking servers used highlighted a family of poses satisfying our criteria. This contained solutions ranked 1, 4 and 5 from ZDOCK, the second solution from GRAMM-X and the third from ClusPro ( Supplementary figure 1).

As shown in Supplementary figure 1, these poses occlude the catalytic site cleft of the enzyme by inserting the conserved IPI wedge. They vary slightly in their rotation about an axis running along the cleft, and to a minor extent in their translation along it, but can be considered as a cluster of solutions.

**Figure 3.**
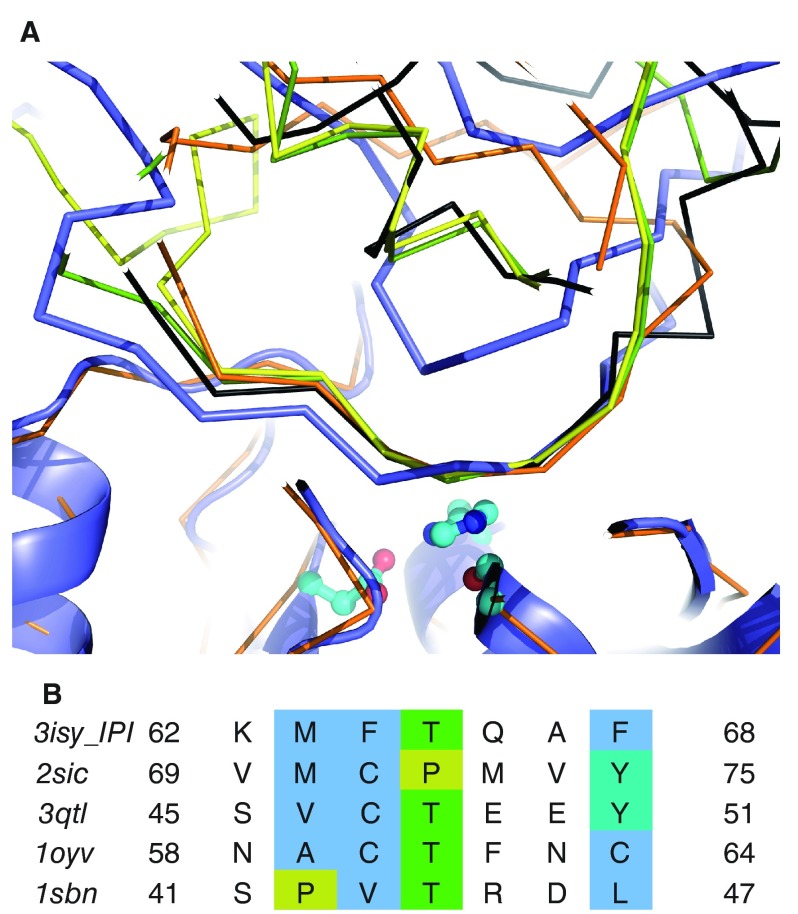
Comparison of Intracellular Protease Inhibitor (IPI) docked to a model of *B. subtilis* subtilisin with other subtilisin-inhibitor complexes. **A**) Inhibitors are drawn as ribbon and the *B. subtilis* subtilisin model as cartoon, with catalytic triad shown as ball-and-stick. The IPI complex is shown in mauve, others, after superposition on the enzymes structure, as green ( 2SIC; *Streptomyces* Subtilisin inhibitor), yellow ( 3QTL; Kazal inhibitor), black ( 1OYV; plant inhibitor class) or orange ( 1SBN; eglin inhibitor class). The inhibitory region lies immediately above the catalytic site in this view. **B**) Comparison of inhibitory region sequences from IPI and four distinct inhibitor classes. The sequences are derived from the structures shown in **A**) and coloured in Jalview ( http://www.jalview.org/ ^44^) using the ClustalX scheme ^45^.

**Figure 4.**
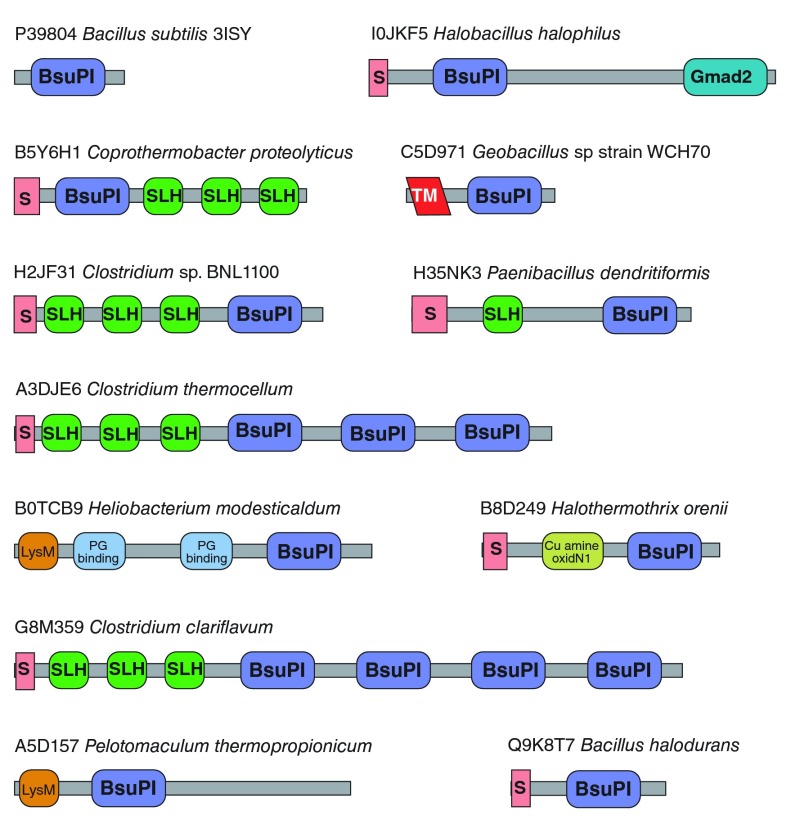
Domain architectures of proteins containing a domain homologous to Intracellular Protease Inhibitor (IPI). Key to domains: BsuPI, *Bacillus subtilis* protein inhibitor-like; S, signal peptide; Gmad2, immunoglobulin-like domain of bacterial spore germination; SLH, S-layer homology domain; TM, transmembrane helix; PG binding, peptidoglycan-binding domain; Cu amine oxidN1, copper amine oxidase N-terminal domain; LysM, lysin motif domain.

It is well known that inhibitors typically inhibit cognate enzymes through interaction of extended regions ^36^ and, furthermore, that different families of inhibitors acting on the same class of enzyme can exhibit highly similar, but convergently evolved interaction features ^36– 38^. Remarkably, we find that the region at the edge of the IPI wedge structure could interact with subtilisin in a manner strongly reminiscent of inhibition modes of other subtilisin family inhibitors. As Figure 3A shows, in a representative of the cluster of docking solutions, solution 5 from ZDOCK, residues 62–68 of IPI superimpose well on strongly similar inhibitory stretches from five distinct inhibitor classes, especially considering the potential for small conformational changes on binding and the fact that a model was used for the docking. Incidentally, the experimentally observed propeptide ^4^, while lying across the catalytic site cleft in the same direction, is different to both IPI and the other four inhibitors in Figure 4.

The comparison between the matching inhibitory regions also revealed suggestive sequence similarities with IPI. As shown in Figure 3B, amino acids in IPI abide by trends evident in the other inhibitors. Most notably three of four previous inhibitors have a central Thr residue, also present in IPI. The following position is the P1 residue. Ordinarily, substrate specificity would dictate conservation here among inhibitors but, as Figure 3B shows, the sequence varies surprisingly at this position, including a Glu in 3QTL, similar to the Gln borne at this position by IPI. The previous two positions and the last are conserved hydrophobic in all sequences; again IPI conforms to the consensus.

### Sequence and structure similarities

IPI homologues are found in both bacteria and archaea, but no homologues have been detected in eukaryotes. All bacterial homologues are from Gram-positive bacteria (Firmicutes), with the exception of homologues from the Gemmatimonadetes bacterium *Gemmatimonas aurantiaca*, and the Gammaproteobacteria *Shewanella oneidensis* and *Kangiella koreensis*. All three of these organisms also possess subtilisin homologues. A subtilisin homologue from *Gemmatimonas aurantiaca* (UniProtKB C1AD56) is predicted to be intracellular.

All of the 30 archaean homologues are from species of the family *Halobacteriaceae*, which includes high salt tolerant species. The archaeans with completely sequenced genomes that possess an IPI homologue also possess at least one subtilisin homologue, which in halophilic archaeon DL31 and *Haloterrigena turkmenica* include proteins predicted to be intracellular. In none of the species with completely sequenced genomes is the gene for the IPI homologue in close proximity to a gene for a subtilisin homologue, and so they are not part of the same operon or regulon. In total, there are 33 species of bacteria and archaea that possess both an IPI homologue and a subtilisin homologue that is predicted to be intracellular (from absence of a signal peptide). There are 43 species from which an IPI homologue is known, but an intracellular subtilisin homologue is unknown, but of these 43 species only four have had their genomes completely sequenced ( *Bacillus selenitireducens*, *Bacillus macauensis*, *Clostridium* sp. BNL1100 and *Selenomonas* sp. oral taxon 137).

It is relatively common for inhibitors to occur as multiple domains within the same protein. Figure 4 shows examples of domain architectures seen in proteins bearing an IPI-like domain. Most of the 109 proteins with IPI-like domains share the simple domain architecture of the *Bacillus subtilis* protein. The presence of a signal peptide shows that many members of the family are secreted rather than intracellular proteins. Even among the proteins that contain only the IPI-like domain, a large number also possess signal peptides, including homologues from *Bacillus* species. One homologue from a *Geobacillus* species is predicted to be a type II membrane protein with an N-terminal transmembrane domain. The longest protein to include an IPI-like domain is the 585-residue Chte0880 protein from *Clostridium thermocellum*, which has three repeats each composed of an S-layer homology (SLH) domain followed by an IPI-like domain. The presence of SLH domains indicates that the protein binds to the proteoglycan of the cell wall.

IPI itself was initially characterized as intracellular. It was purified by lysing cells with lysozyme and its N-terminal sequence determined by Edman degradation ^5^. Later, when the gene was sequenced ^7^, no signal peptide was detected in the coding sequence. Now that the full genome for *Bacillus subtilis* strain 168 has been sequenced ^39^, it is clear that a possible alternative initiating methionine exists for the *ipi* gene and the coding sequence could be extended at the N-terminus, adding an extra in-frame 33 residues (MKRLLVMLLPVLLLIGCGKDEQTEPDKEVSGG). The predicted protein sequences of IPI from *B. subtilis* strains BSn5 ^40^ and QB928 ^41^ include this N-terminal extension. When the extended sequence was submitted to the SignalP ( http://www.cbs.dtu.dk/services/SignalP/) a signal peptide was predicted with cleavage at the Thr23-Glu bond. The N-terminal sequence determined by Nishino *et al.* (1986) ^5^ was Glu34, with the assumption that Met33 was the initiating methionine. Signal peptidase 1 is unlikely to cleave the Met33-Glu bond, because of the hydrophilic nature of the region Glu24-Gly32. This may mean that alternative initiation exists for this protein; a secreted form starting at Met1 and an intracellular form starting at Met33. It is worth adding that even if a majority of IPI is in fact secreted, a protease inhibitor function is still physiologically plausible since most subtilisins are extracellular.

### Is IPI truly a peptidase inhibitor?

The nearest structural neighbours of IPI, including the RbmA protein and coagulation factor XIII, are involved in protein-protein interactions. This may indicate that rather than functioning as an inhibitor, IPI is binding the peptidase in some non-specific way. Such an interaction might explain the peculiar results obtained with the substrate casein, which apparently “repressed” inhibition of the peptidase ^6^. Binding to an intracellular subtilisin may therefore not represent the primary physiological function of IPI. However, there are similarities between the IPI protein and chagasin, and not only in terms of structure. Chagasin inhibition of cysteine peptidases is tight but reversible ^33^. If IPI inhibition of subtilisins were also reversible, then the repression seen with casein would be explained. IPI inhibition would not be via the classical Laskowski mechanism, in which a reactive site bond *permanently* occupies the peptidase active site, but access to the active site would instead be *reversibly* physically blocked.

Without further kinetics studies it is not possible to state categorically that IPI definitely is a peptidase inhibitor. However, the tertiary structure and docking predictions illustrate how it could feasibly inhibit its cognate enzyme. That hypothetical mode bears strong similarities to those seen for other well-characterised, unrelated serine peptidase inhibitor families such as the Kazal and Kunitz groups in agreement with a substantial literature on convergent evolution in peptidase inhibition ^36, 38^. It is intriguing that IPI may exemplify a second intracellular peptidase inhibitor family, after the chagasin and amoebiasin group, with an Ig-type fold. However, structural similarity between chagasin and IPI is weak and the two groups clearly share no recent common ancestor. We hope the resolution of the structure encourages others to further characterize IPI and proteins bearing IPI-like domains to further probe their functions.

## Figures and Tables

**Supplementary table 1.** Crystallographic data and refinement statistics for IPI, BSU11130 (PDB code 3ISY). Values in parentheses are for the highest resolution shell. Space group: I 4 _1_ 2 2. Unit cell parameters: a = 73.6, c = 132.9 Å.

Data collection	λ1 MAD-Se	λ2 MAD-Se
Wavelength (Å)	0.91162	0.97934
Resolution range (Å)	38.0–2.61 (2.75–2.61)	41.0–2.71 (2.86–2.71)
No. of observations	41,044	36,556
No. of unique reflections	5864	5264
Completeness (%)	100 (100)	100 (99.9)
Mean I/σ (I)	18.7 (2.6)	19.0 (3.5)
R _merge_ on I† (%)	9.4 (87.4)	10.6 (63.0)
R _meas_ on I‡ (%)	10.1 (94.2)	11.4 (67.9)
Model and refinement statistics		
Resolution range (Å)	37.96–2.61	
No. of reflections (total)	5851§	
No. of reflections (test)	266	
Completeness (%)	99.9	
Data set used in refinement	λ1	
Cutoff criteria	|F|>0	
R _cryst_¶	0.203	
R _free_¶	0.243	
Stereochemical parameters		
Restraints (r.m.s.d. observed)		
Bond angles (º)	1.521	
Bond lengths (Å)	0.014	
Average isotropic B value†† (Å ^2^)	45.520	
ESU‡‡ based on R _free_ (Å)	0.273	
Protein residues/atoms	117/967	
Waters/solvent molecules	22	

† R _merge_ = ΣhklΣi|Ii(hkl)-(I(hkl))|/Σhkl Σi(hkl). Highest resolution shell in parentheses.

‡ R _meas_ = Σhkl[N/(N-1)]1/2Σi|Ii(hkl)-(I(hkl))|/ΣhklΣiIi(hkl) ^46^.

§ Typically, the number of unique reflections used in refinement is slightly less than the total number that were integrated and scaled. Reflections are excluded owing to negative intensities and rounding errors in the resolution limits and unit-cell parameters.

¶ R _cryst_ = Σhkl||F _obs_|-|F _calc_||/Σhkl|F _obs_|, where F _calc_ and F _obs_ are the calculated and observed structure-factor amplitudes, respectively. R _free_ is the same as R _cryst_ but for 4.9% of the total reflections chosen at random and omitted from refinement.

†† This value represents the total B that includes TLS and residual B components.

‡‡ Estimated overall coordinate error (Collaborative Computational Project, Number 4, 1994; ^47^).

**Supplementary table 2.** Comparison of the secondary structural elements assigned by Li *et al.* ^16^ by NMR and those observed in the crystal structure ( Figure 2A).

Secondary structure element (Li *et al.* ^16^)	Residue range (NMR)	Residue range (crystal structure)
β-1	6–12	5–15
β-2	18–27	18–28
β-3	32–37	33–37
β-4	43–48	42–49
β-5	55–57	53–58
β-6	62–65	*not seen*
β-7	68–73	69–73
β-8	78–86	77–85
β-9	93–100	92–101
β-10	103–104	103–105
β-11	109–118	110–118
