# Supplementary material for: The first structure in a family of peptidase inhibitors reveals an unusual Ig-like fold
Source: F1000Res. 2013 Aug 23;2:154. Originally published 2013 Jul 10. [Version 2] doi: 10.12688/f1000research.2-154.v2 (PMC3901451; doi:10.12688/f1000research.2-154.v2)
Supplement: Validation report for PDB: 3ISY — This is the first structure in a family of peptidase inhibitors derived from Bacillus subtilis. The structure has an unusual Ig-like fold. [file f1000research-2-2157-s0000.tgz › Validation_report_3isy.pdf]

The following geometrical and stereochemical features have been calculated for your structure.

CLOSE CONTACTS

==> Close contacts in same asymmetric unit. Distances smaller than 2.2 Angstroms are considered as close contacts.

none

==> Close contacts based on crystal symmetry. Distances smaller than 2.2 Angstroms are considered as close contacts.

none

BOND DISTANCES AND ANGLES

==> Bond and angle checks are performed by first computing the average rms error for all bonds and angles relative to standard values for nucleotide units [L. Clowney et al., Geometric Parameters in Nucleic Acids: Nitrogenous Bases, J.Am.Chem.Soc. 1996, 118, 509-518; A. Gelbin et al., Geometric Parameters in Nucleic Acids: Sugar and Phosphate Constituents, J.Am.Chem.Soc. 1996, 118, 519-529] and amino acid units [R.A. Engh and R. Huber, Structure quality and target parameters, International Tables for Crystallography, Volume F, 2001, 382-392]. Any bond or angle which deviates from the dictionary values by more than six times this computed rms error is identified as an outlier.

*** Covalent Bond Lengths:

The overall RMS deviation for covalent bonds relative to the standard dictionary is 0.014 Angstroms

All covalent bonds lie within a 6.0*RMSD range about the standard dictionary values.

*** Covalent Angle Values:

The overall RMS deviation for covalent angles relative to the standard dictionary is 1.6 degrees.

All covalent bond angles lie within a 6.0*RMSD range about the standard dictionary values.

TORSION ANGLES

The torsion angle distributions have been checked. To view these reports, please refer to the ADIT Validation Server at http://pdb.rutgers.edu/validate.

==> The following table contains a list of torsion angles outside the expected Ramachandran regions [GJ. Kleywegt and TA. Jones, PHI/PSI-chology: Ramachandran Revisited, Structure 1996, 4, 1395 - 1400].

Residue	Chain	Sequence	PSI	PHI
LYS	A	86	37.67	-90.96
GLU	A	87	118.55	-163.23

CHIRALITY

The chirality has been checked. O1P, O2P, and hydrogen atoms which do not follow the convention defined in the IUBMB (Liebecq, C. Compendium of Biochemical Nomenclature and Related Documents, 2nd ed.; Portland Press: London and Chapel Hill, 1992) and IUPAC nomenclature (J.L. Markley, A. Bax, Y. Arata, C.W. Hilbers, R. Kaptein, B.D. Sykes, P.E. Wright and K. Wuthrich, Recommendations for the Presentation of NMR Structures of Proteins and Nucleic Acids, Pure & Appl. Chem., Vol. 70, pp. 117-142, 1998) have been standardized. Any other stereochemical violations are listed below.

SOLVENT

The following solvent molecules are further than 3.5 Angstroms away from macromolecule atoms in the asymmetric unit that are available for hydrogen bonding. Solvent molecules in extended hydration shells separated by 3.5 Angstroms or less are not listed.

none

We have replaced the coordinates for solvent molecules which could be translated back into the asymmetric unit. Please review all solvent molecules in your file and contact us if you have any serious objections.

MISSING RESIDUES

==> The following residues are missing:
(Note: The SEQ number starts from 1 for each chain according to SEQRES sequence record.)

```
 RES MOD#C SEQ

 GLY(    A   0 )
 MSE(    A   1 )
 GLU(    A   2 )


PDB Chain_ID: A

        1                                                   15
SEQRES: GLY MSE GLU ASN GLN GLU VAL VAL LEU SER ILE ASP ALA ILE GLN
COORDS:   ?   ?   ? ASN GLN GLU VAL VAL LEU SER ILE ASP ALA ILE GLN
                    3                                       14

        16                                                  30
SEQRES: GLU PRO GLU GLN ILE LYS PHE ASN MSE SER LEU LYS ASN GLN SER
COORDS: GLU PRO GLU GLN ILE LYS PHE ASN MSE SER LEU LYS ASN GLN SER
        15                                                  29

        31                                                  45
SEQRES: GLU ARG ALA ILE GLU PHE GLN PHE SER THR GLY GLN LYS PHE GLU
COORDS: GLU ARG ALA ILE GLU PHE GLN PHE SER THR GLY GLN LYS PHE GLU
        30                                                  44

        46                                                  60
SEQRES: LEU VAL VAL TYR ASP SER GLU HIS LYS GLU ARG TYR ARG TYR SER
COORDS: LEU VAL VAL TYR ASP SER GLU HIS LYS GLU ARG TYR ARG TYR SER
        45                                                  59

        61                                                  75
SEQRES: LYS GLU LYS MSE PHE THR GLN ALA PHE GLN ASN LEU THR LEU GLU
COORDS: LYS GLU LYS MSE PHE THR GLN ALA PHE GLN ASN LEU THR LEU GLU
        60                                                  74

        76                                                  90
SEQRES: SER GLY GLU THR TYR ASP PHE SER ASP VAL TRP LYS GLU VAL PRO
```

```
   COORDS: SER GLY GLU THR TYR ASP PHE SER ASP VAL TRP LYS GLU VAL PRO
           75                                                       89

           91                                                      105
   SEQRES: GLU PRO GLY THR TYR GLU VAL LYS VAL THR PHE LYS GLY ARG ALA
   COORDS: GLU PRO GLY THR TYR GLU VAL LYS VAL THR PHE LYS GLY ARG ALA
           90                                                      104

           106                                                     120
   SEQRES: GLU ASN LEU LYS GLN VAL GLN ALA VAL GLN GLN PHE GLU VAL LYS
   COORDS: GLU ASN LEU LYS GLN VAL GLN ALA VAL GLN GLN PHE GLU VAL LYS
           105                                                     119
```

MISSING ATOMS

```
==> The following residues have missing atoms:

     RES MOD#C SEQ          ATOMS

     GLU(    A  17)          CG   CD  OE1  OE2
     LYS(    A  60)          CD   CE   NZ
     LYS(    A  97)          CE   NZ
```

Sequence Identity between entity 1 and target 392216 = 99%
