# Supplementary material for: The first structure in a family of peptidase inhibitors reveals an unusual Ig-like fold
Source: F1000Res. 2013 Aug 23;2:154. Originally published 2013 Jul 10. [Version 2] doi: 10.12688/f1000research.2-154.v2 (PMC3901451; doi:10.12688/f1000research.2-154.v2)
Supplement: Peer review history

# Peer review: Referee response for version 2

**DOI:** 10.5256/f1000research.2157.r3278

**Published:** 2014-01-22

**Authors:** Patricia C Weber

**Affiliations:** 1 Imiplex LLC, Bristol, PA, USA

I have read this submission. I believe that I have an appropriate level of expertise to confirm that it is of an acceptable scientific standard.

# Peer review: Referee response for version 2

**DOI:** 10.5256/f1000research.2157.r1733

**Published:** 2013-09-09

**Authors:** Xiayang Qiu

**Affiliations:** 1 Department of Structural Biology, Pfizer Inc, Groton, CT, USA

Looks good.

I have read this submission. I believe that I have an appropriate level of expertise to confirm that it is of an acceptable scientific standard.

# Peer review: Referee response for version 1

**DOI:** 10.5256/f1000research.1790.r1142

**Published:** 2013-07-29

**Authors:** Xiayang Qiu

**Affiliations:** 1 Department of Structural Biology, Pfizer Inc, Groton, CT, USA

This article reports the first crystal structure of the IPI protein from *B. subtilis*, a family of protease inhibitor proteins with homologues across many different species. Although being assigned to the general IG-like fold, the structure shows a distinct topology, hence could also be regarded as a novel fold and a significant contribution to the protease inhibitor field. Additional studies are needed to understand the functional aspects of this new protein and structural scaffold.

I have read this submission. I believe that I have an appropriate level of expertise to confirm that it is of an acceptable scientific standard.

# Peer review: Referee response for version 1

**DOI:** 10.5256/f1000research.1790.r1144

**Published:** 2013-07-29

**Authors:** Patricia C Weber

**Affiliations:** 1 Imiplex LLC, Bristol, PA, USA

Thorough discussion of the inhibition mechanism and the protein's possible biological role(s) make this manuscript of interest to a wide audience.  

Minor changes to improve clarity include delaying (or removing) discussions of ISP in the Abstract because ISP is not the central subject of the manuscript, expanding comments on the NMR structure and possibly citing similarities beyond the common identification of beta strands, and fixing Figure 1A where greater separation between the stereo images is needed.

I have read this submission. I believe that I have an appropriate level of expertise to confirm that it is of an acceptable scientific standard.
